# Identification of candidate HLA-E binding peptides from *Mycobacterium tuberculosis* using phage secretome library screening and computational modeling

**DOI:** 10.3389/fcimb.2026.1724374

**Published:** 2026-10-21

**Authors:** Nkanyezi Masondo, Michelle Lucille Gordon, Thamsanqa Emmanuel Chiliza

**Affiliations:** 1 School of Laboratory Medicine and Medical Sciences, College of Health Sciences, University of KwaZulu-Natal, Durban, KwaZulu-Natal, South Africa; 2 Discipline of Microbiology, School of Agriculture and Science, College of Agriculture, Engineering and Science, University of KwaZulu-Natal, Durban, KwaZulu-Natal, South Africa; 3 Igagasi Biotech (Pty) Ltd, TIA Bioprocessing Platform, Durban, KwaZulu-Natal, South Africa

**Keywords:** HLA-E, molecular docking, *Mycobacterium tuberculosis*, phage display, secretome

## Abstract

**Background:**

*Mycobacterium tuberculosis* (*Mtb*) remains a primary global health challenge, particularly in HIV-co-infected populations where viral Nef-mediated downregulation of classical MHC-I molecules impairs T-cell immunity. Conversely, the non-classical MHC-Ib molecule, HLA-E, is resistant to Nef-mediated evasion, representing an attractive, non-polymorphic pathway for investigating potential *Mtb*-specific CD8^+^ T-cell activation targets.

**Methods:**

This study utilized an exploratory *in vitro* screening and *in silico* modeling framework to identify candidate HLA-E-binding *Mtb* peptides. Candidate ligands were screened by biopanning an *Mtb* F15/LAM4/KZN phage secretome library against immobilized recombinant HLA-E*01:03. Identified peptides were characterized via DNA sequencing, and their predicted structural–mechanical interactions were evaluated using molecular docking and normal mode analysis (NMA).

**Results:**

Phage selection yielded distinct *Mtb*-derived peptide fragments, including components from known immunogenic antigens Rv1980c (Mpt64) and Rv2875 (Mpt70), alongside conserved hypothetical proteins such as Rv1566c. Structural profiling predicted groove-directed anchoring within the modeled HLA-E binding cleft, displaying a spatial preference for pockets E and F. Computational molecular scoring prioritized favorable candidates (e.g., Rv2972c at −87.49 kcal/mol), while NMA indicated that these prioritized *Mtb* peptides possess a higher degree of predicted conformational plasticity (eigenvalues as low as 9.13 × 10^-6^) compared to the rigid Mtb44 baseline (1.37 × 10^–4^).

**Conclusion:**

These findings map an exploratory *Mtb* peptide repertoire demonstrating computational feasibility for flexible HLA-E groove accommodation. By providing a structural foundation for candidate peptide prioritization, these results lay the groundwork for future investigations into unconventional CD8^+^ T-cell activation and the rational design of next-generation TB vaccines tailored for high-burden TB/HIV co-infection settings.

## 1 Introduction

Tuberculosis (TB) remains a major global health threat, causing high mortality rates despite the widespread availability of treatments, with an estimated 10.7 million new cases occurring annually. While steady progress has been made in reducing overall incidence and mortality, accelerated efforts are required to meet the World Health Organization’s (WHO) End TB Strategy goal by 2035 (World Health Organization, 2024; Goletti et al., 2025). A more effective vaccine is urgently needed to combat this global epidemic, as the current Bacille Calmette-Guérin (BCG) vaccine offers highly variable and limited protection, particularly against pulmonary TB in adults. Notably, most individuals infected with *Mycobacterium tuberculosis* (*Mtb*) remain asymptomatic, indicating that control of the pathogen is largely mediated by the cellular arm of the host immune system (Lange et al., 2022).

The host immune system plays a crucial role in controlling *Mtb* infection, with both innate and adaptive immunity contributing to protection. Cell-mediated immune resistance to *Mtb* primarily involves CD4^+^ and CD8^+^ T cells. Major histocompatibility complex (MHC) class II molecules (HLA-DR, HLA-DQ, HLA-DP) present extracellular antigens on the surface of professional antigen-presenting cells (APCs) such as dendritic cells (DCs), B cells, and macrophages to CD4^+^ T cells (Benvenuti, 2016; Paterson et al., 2024). In contrast, classical MHC class I molecules (HLA-A, HLA-B, HLA-C) present endogenous antigens to CD8^+^ T cells (Moreau and Bousso, 2014). The human MHC class I family also encompasses the non-classical MHC class Ib molecules, which include HLA-E, HLA-F, and HLA-G (Cruz et al., 2017).

While the roles of classical MHC molecules and their corresponding T-cell subsets are well-established, an emerging focus in structural immunology is the function of the non-classical molecule, HLA-E. Non-classical MHC molecules exhibit minimal genetic polymorphism and fulfill specialized immunoregulatory roles. In innate immunity, HLA-E presents signal sequence-derived peptides from classical HLA class I leader sequences to CD94/NKG2 receptors on natural killer (NK) cells, thereby inhibiting NK-mediated cytotoxicity and maintaining self-tolerance (Braud et al., 1998; Lee et al., 1998; Kraemer et al., 2014). Crucially, while human immunodeficiency virus (HIV-1) downregulates the expression of classical HLA-A and HLA-B via its Nef accessory protein to evade host surveillance, HLA-E is resistant to this Nef-mediated downregulation due to specific amino acid substitutions in its cytoplasmic tail. Consequently, HLA-E-dependent antigen presentation remains fully functional during HIV-1/*Mtb* co-infection, an immunological reality highly prevalent in TB-endemic regions (Grimsley et al., 2002; Caccamo et al., 2015; Joosten et al., 2016; McMurtrey et al., 2017; Hannoun et al., 2018; Prezzemolo et al., 2018).

Accumulating evidence demonstrates that HLA-E can bind and present pathogen-derived antigens, including those from *Mtb* to conventional and unconventional CD8^+^ T cells, thereby driving adaptive immunity (Lewinsohn et al., 1998; Heinzel et al., 2002; Joosten et al., 2010; Caccamo et al., 2015; van Meijgaarden et al., 2015; Hannoun et al., 2018). This unconventional presentation paradigm positions HLA-E as an exceptionally attractive candidate for a universal TB vaccine strategy capable of eliciting broad immunity across genetically diverse global populations (Joosten et al., 2019; Grant et al., 2020). Previous studies have successfully identified HLA-E-restricted *Mtb* peptides using bioinformatics prediction pipelines and *in vitro* binding assays. For instance, Joosten et al. used computational screening paired with *in vitro* stabilization assays to identify 69 candidate peptides; of these, 36 demonstrated measurable binding to HLA-E, 18 exhibited high binding affinity, and 11 successfully activated CD8^+^ T cells (Joosten et al., 2010). Similarly, mass spectrometry-based immunopeptidomics has identified 28 HLA-E ligands derived from 13 distinct *Mtb* proteins, revealing epitopes that are broadly recognized by CD8^+^ T cells from patients with active TB, latent infection, and healthy controls (Harriff et al., 2017; McMurtrey et al., 2017). However, traditional whole-cell immunopeptidomics methods are frequently biased toward highly abundant intracellular or structural proteins, often failing to capture low-abundance, transiently expressed secreted factors that drive early host–pathogen interactions. Consequently, the full antigenic repertoire of HLA-E-restricted *Mtb* peptides remains incompletely defined (Grant et al., 2020), with the vast majority of secretome-derived targets remaining uncharacterized.

To address this critical gap, the present study deployed phage display technology to select and map *Mtb* peptides displaying binding affinity for the HLA-E molecule. Phage display represents a robust methodology for screening massive, diverse peptide libraries against immobilized molecular targets through cycles of target binding, high-stringency washing to eliminate non-specific interactors, and elution of high-affinity binders (Smith, 1985).

In this work, an *Mtb* F15/LAM4/KZN phage secretome library was screened against the recombinant extracellular domain of the HLA-E receptor. This specialized secretome library derived from the genomic DNA of the clinically relevant, extensively drug-resistant (XDR) *Mtb* F15/LAM4/KZN strain specifically displays secreted proteins on the surface of bacteriophages (Chiliza et al., 2017). Because the *Mtb* secretome comprises the primary interface interacting with the host environment and cellular mechanisms, this library serves as an ideal repository for identifying candidate biomarkers for novel subunit vaccines, diagnostics, and therapeutic targets (Chiliza et al., 2019). Characterizing HLA-E-binding peptides from this secretome provides novel insights into the molecular interaction profile of *Mtb*, establishing a critical baseline for computational prioritization and a structural biology framework. Given the established role of HLA-E in antigen presentation pathways, the identification of these novel *Mtb* peptides expands the known structural interaction repertoire. These findings, derived from our targeted screening, provide a foundational platform to guide subsequent experimental characterization, downstream validation, and translational evaluation.

## 2 Materials and methods

### 2.1 Bacterial strain, phage display library, and protein

The *Mtb* F15/LAM4/KZN phage secretome library previously constructed was used in the study (Chiliza et al., 2017). Briefly, the construction of the *M. tuberculosis* F15/LAM4/KZN phage secretome sublibrary, as described by Chiliza et al. (2017) based on the protocol by Jankovic et al. (2007), relies on a “survival-of-the-fittest” structural selection to filter out non-exported proteins from the whole genome. First, the *Mtb* whole genomic library in the pDJ01 phagemid vector is rescued with helper phage to produce physical particles. Because the pDJ01 system requires a functional bacterial signal peptide to correctly assemble the phage cap, only clones containing true secretory inserts form robust, intact particles. When the mixture is treated with the detergent sarcosyl and DNase I, non-secretory clones, which are structurally defective and unstable, are lysed and their DNA is digested. The remaining, resilient secretome phagemids are then lysed to release their single-stranded DNA (ssDNA), which is purified via phenol–chloroform extraction and transformed into *Escherichia coli* TG-1 to yield the final 8 × 10^3^ *Mtb* F15/LAM4/KZN phage secretome sublibrary variants (Chiliza et al., 2017).

Recombinant human HLA-E heavy chain ectodomain was purchased from Cusabio Technology LLC (Houston, TX, USA; Cat. No. CSB-EP320269HU). The construct encodes a monomeric, partial segment of the mature HLA-E α-chain (corresponding to the HLA-E*01:03 allele, UniProt P13747) spanning residues 22–305 of the extracellular region.

### 2.2 *Mtb* phage library screening against immobilized recombinant HLA-E protein

An ELISA Nunc MaxiSorp microtiter plate (Thermo Fisher Scientific, Waltham, MA, USA) was coated with 10 µg/mL of recombinant HLA-E protein overnight at 4 °C. All subsequent panning steps were performed at room temperature. The target wells were blocked for 1 h with 2% fat-free milk powder (MP) in 1× PBS buffer, followed by three washes with PBS. Concurrently, to eliminate non-specific background and plastic-binding artifacts, a subtractive prescreening step was executed: the *Mtb* phage secretome library aliquot was preincubated for 30 min in 2% MP within separate, uncoated blank microtiter wells. This precleared phage supernatant was then transferred to the target HLA-E-coated wells and incubated stationarily for 90 min. Following incubation, the wells were washed 10× with PBS containing 0.1% Tween 20 (PBS/T) and rinsed an additional 10× with PBS. Bound phages were eluted with 150 µL of 100 mM triethylamine (pH 12) for 10 min and immediately neutralized by adding 75 µL of 1 M Tris–HCl (pH 7.4). The neutralized phages were used to reinfect exponentially growing *E. coli* TG1 cells before plating on TYE plates containing 2% glucose and 100 µg/mL chloramphenicol. Colonies were harvested following overnight incubation at 30°C (Chiliza et al., 2019).

### 2.3 PCR amplification and Sanger sequencing of DNA inserts

Bacterial colonies were inoculated into 5 mL of 2×TY broth supplemented with chloramphenicol and glucose (2×TY C/G) and cultured overnight at 30 °C with shaking at 250 rpm. Phagemid DNA was extracted from the overnight cultures using the Zyppy Plasmid Miniprep Kit (Zymo Research Corporation, Irvine, CA, USA) according to the manufacturer’s instructions. To verify and amplify the inserted fragments, colony PCR was executed using pDJ01 forward (5′-ATGTTGCTGTTCTTCA-3′) and pDJ01 reverse (5′-CCGGAAACGTCACCAATGAA-3′) primers (Jankovic et al., 2007). The PCR conditions comprised an initial denaturation at 94 °C for 5 min; followed by 30 cycles of denaturation at 95 °C for 30 s, annealing at 52 °C for 30 s, and extension at 72 °C for 1 min; concluding with a final extension phase at 72 °C for 5 min. PCR amplicons were submitted to Inqaba Biotechnical Industries (Pty) Ltd (Pretoria, South Africa) for bidirectional DNA sequencing. The resulting sequence chromatograms were analyzed, trimmed, and aligned using the BioEdit Sequence Alignment Editor (Hall, 2011).

### 2.4 Bioinformatic identification and functional classification of *Mtb* proteins

Nucleotide sequences obtained from the sequenced phagemid inserts were analyzed using the BLASTn algorithm against the *Mtb* comparative database within the Bacterial and Viral Bioinformatics Resource Center (BV-BRC, formerly PATRIC; https://www.bv-brc.org/) to identify the corresponding genes and their encoded proteins (Davis et al., 2020). Following identification, the Mycobrowser database (https://mycobrowser.epfl.ch/) was used to systematically categorize each validated *Mtb* protein into its respective functional categories (Kapopoulou et al., 2011).

### 2.5 *In Silico* modeling and structural coordination of HLA-E and *Mtb* peptides

Three-dimensional structural models for both the non-classical receptor molecule and the selected *Mtb* peptide ligands were generated using the Phyre2 (http://www.sbg.bio.ic.ac.uk/phyre2) web server framework, which executes remote homology detection and intensive fold recognition protocols (Powell et al., 2025). To ensure the reliability of the monomeric HLA-E structure used for molecular docking, a comprehensive structural validation was performed using the MolProbity web server (http://molprobity.biochem.duke.edu/) (Williams et al., 2018). This analysis evaluated global model quality and assessed local precision through Ramachandran plot distribution, rotamer analysis, and clash scores, ensuring that the peptide-binding interface architecture remained consistent with high-resolution structural standards. The primary structural template for the receptor was modeled using the definitive primary amino acid sequence of the human HLA-E*01:03 allelic variant (UniProt ID: P13747), while the corresponding 3D structures for the prioritized *Mtb* hit fragments were modeled from their respective translated peptide sequences.

### 2.6 Analysis of HLA-E:Mtb peptide interactions via molecular docking

Molecular docking simulations were executed using the PatchDock server (https://bio3d.cs.huji.ac.il/webserver/patchdock/), designating the modeled HLA-E*01:03 structure as the receptor and the prioritized *Mtb* peptide geometries as the ligands. PatchDock utilizes a geometry-based docking algorithm that optimizes molecular shape complementarity by partitioning structural surfaces into concave, convex, and flat patches to generate candidate docking conformations (Huang et al., 2010). The top-ranked candidate complexes were systematically transferred into the FireDock server (http://bioinfo3d.cs.tau.ac.il/FireDock/) for high-throughput refinement and side-chain optimization (Andrusier et al., 2007). FireDock concurrently addresses interface flexibility and binding energy scoring across the top 1,000 rigid-body solution candidates. The resulting refined complexes were sorted and prioritized based on their calculated global docking score functions. Structural visualization, spatial alignment, and localized intermolecular interaction analyses of the final docked complexes were performed using PyMOL (Mashiach et al., 2008).

### 2.7 Normal mode analysis and structural dynamics

To evaluate the conformational dynamics and mechanical plasticity of our HLA-E scaffold, we employed normal mode analysis (NMA). Given that the candidate peptides identified in this study are significantly extended, ranging from 32 to 85 amino acids, these solvent-exposed segments present a complex structural landscape that requires a focus on global architecture. NMA, by utilizing internal coordinates, is uniquely suited for characterizing these systems as it effectively isolates large-scale, low-frequency collective motions from localized thermal fluctuations. This approach allows us to derive a robust mechanical profile that reflects the predicted structural adaptability critical to our hypothesis. By focusing on these global vibrational modes, we gain essential insight into the predicted conformational behavior and dynamic responsiveness of the binding groove when engaged with these novel, non-canonical ligands.

To facilitate this assessment, we used the iMODS server (https://imods.chaconlab.org/), which is specifically designed to perform NMA using internal coordinates (López-Blanco et al., 2014). This computational approach explores the large-scale functional dynamics and conformational plasticity of the protein interfaces. The refined PDB files of the top-ranked docking complexes were utilized as input. The simulations were executed using the server’s default parameters, which characterize structural dynamics through several key metrics: (1) eigenvalues (lambda)—used to quantify the energy required to deform the structure; lower values indicate higher conformational flexibility and a lower energy barrier for structural deformation; (2) deformability—calculated to identify specific residues susceptible to structural distortion within the binding groove; (3) B-factors—derived from NMA to estimate the relative mobility and atomic-level fluctuations of the complex; and (4) covariance matrix and elastic networks—employed to map coordinated movements and atomic-level linkages between the HLA-E receptor and the peptide backbone. This dynamic profiling characterized the predicted conformational flexibility and collective atomic motions of the modeled complexes (López-Blanco et al., 2014). The known HLA-E-restricted epitope Mtb44 (RLPAKAPLL) complex (PDB DOI: https://doi.org/10.2210/pdb6GH4/pdb) was utilized specifically as a structural benchmark for NMA dynamics. Global docking score calculations were omitted for this control sequence, as its 9-mer length fell below the structural configuration thresholds optimized for our primary target candidate docking pipeline.

## 3 Results

### 3.1 Phage display screening and selection of *Mtb* antigens

To identify *Mtb* peptides capable of binding the HLA-E receptor, an *Mtb* phage secretome library was screened against the recombinant HLA-E 01:03 ectodomain (CSB-EP320269HU). A single round of biopanning against immobilized HLA-E followed by propagation of eluted phages in *E. coli* resulted in 36 colonies. Colony PCR confirmed the presence of *Mtb* insert DNA with amplicon bands of varying sizes (**Figure 1**). Subsequent phage DNA sequencing and BLAST analysis against *Mtb* databases revealed 30 unique sequences encoding 30 different *Mtb* protein peptides (**Supplementary Table 1**).

**Figure 1:**
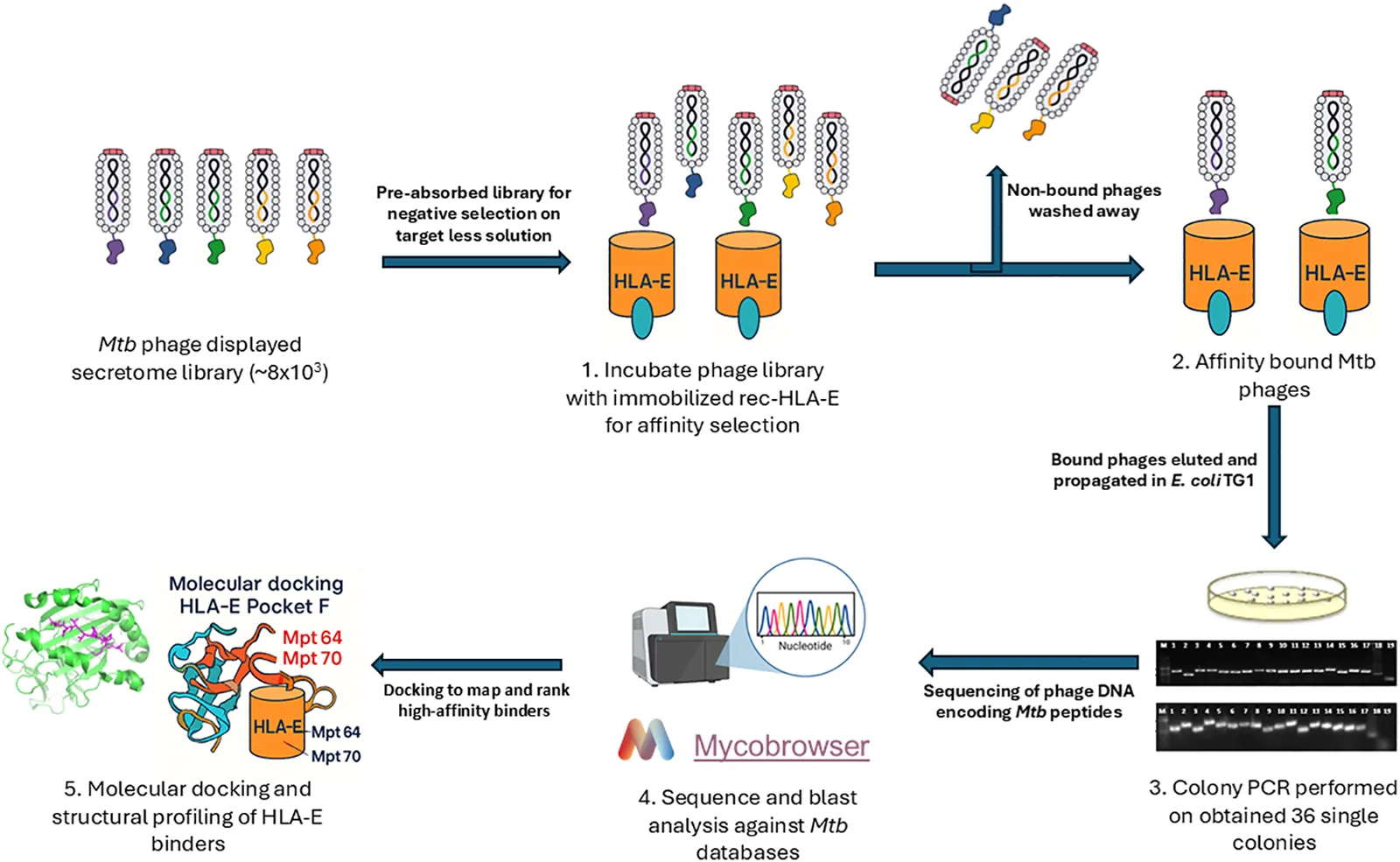
Schematic workflow of the hybrid experimental and computational pipeline for identifying novel HLA-E-binding *Mtb* peptides. The workflow comprises (1) enrichment selection (biopanning) of an *Mtb* phage-displayed secretome library (8 × 10^3^) against immobilized recombinant HLA-E molecules; (2) systematic washing to remove non-bound phages, while specifically bound phages are retained, eluted, and propagated in *E. coli* TG1; (3) confirmation of successful enrichment and the presence of *Mtb* DNA inserts via colony PCR performed on single colonies displaying distinct amplicon bands; (4) phage DNA sequencing, sequence analysis, and BLAST alignment against the Mycobrowser and BV-BRC (formerly PATRIC) databases to reveal unique *Mtb* protein-derived peptides; and (5) subjecting the identified non-canonical *Mtb* peptide sequences to molecular docking to map localized predicted binding footprints such as those belonging to major secreted antigens Mpt64 and Mpt70 nested within specific receptor domains like the HLA-E F pocket.

Because the commercial recombinant construct represents a monomeric, partial HLA-E α-chain isolated from β-2-microglobulin (β2m), it lacks the fully coordinated quaternary conformation of a native trimer. To rigorously address this structural limitation and minimize false-positive phage selection, a negative selection by preincubation of phages on the panning matrix without HLA-E was implemented to deplete the library of phages with non-specific affinity, and stringent washing parameters were implemented during the biopanning cycle. This selection bottleneck acted as a functional filter, ensuring that stable interactors capable of surviving the prescribed buffer conditions remained attached to the ectodomain framework for subsequent downstream computational validation.

### 3.2 Molecular docking and predictive structural profiling of HLA-E binders

Molecular docking is a well-established *in silico* method used to predict the optimal orientation and global docking score of a ligand within the binding pocket of a target receptor, evaluating potential structural complementarity (Pinzi and Rastelli, 2019). In this study, structural macromolecular models of both the identified *Mtb* peptides and the HLA-E receptor were generated using the Phyre2 web server prior to docking simulations. It is important to note that four peptides (Rv3452, Rv2410c, Rv3392c, and Rv1157c) and Mtb44 (control sequence) were excluded from this analysis, as their sequence lengths were below the 30-amino acid threshold required for successful modeling by the Phyre2 server.

To evaluate the structural suitability of the monomeric HLA-E model for *Mtb*-derived peptide docking simulations, we performed a quality assessment using the MolProbity server. The model achieved a MolProbity score of 1.78 (86th percentile), consistent with established structural quality benchmarks. Detailed validation metrics are provided in **Supplementary Table 2**; **Supplementary Figure 1**; specifically, 97.79% of residues occupy favored regions with 0% Ramachandran outliers, reflecting acceptable backbone geometry. Furthermore, a Rama *Z*-score of 0.21 ± 0.45 indicates that the backbone dihedral angles align with expected distributions (Sobolev et al., 2020). These data suggest that the peptide-binding pocket possesses geometric features suitable for ligand interaction modeling, supporting the application of this monomeric scaffold within the simulation platform.

Consequently, macromolecular docking was utilized to predict the atomic-level geometry of the protein–peptide complexes (Zhang et al., 2022) and to evaluate the potential of the remaining 26 full-length *Mtb*-derived peptides to bind within the HLA-E receptor groove. Based on the predicted structural profiling detailed in **Table 1**, the sequence lengths of this candidate pool range from 32 to 85 amino acids, substantially exceeding the classical 9-mer paradigm typical of canonical HLA-E signal peptides (such as the VL9 motif) (Braud et al., 1997; Lee et al., 1998; Voogd et al., 2025). By utilizing full-length modeled structures rather than truncated strings, this macromolecular docking approach allowed for the prediction of localized, core binding footprints nested within the larger, non-canonical peptide architectures.

**Table 1:** Functional annotation, sequence mapping, and site-specific pocket interaction networks of HLA-E*01:03-selected *M. tuberculosis* peptide fragments.

Functional category	Rv name	Protein	AA position	Mtb peptide sequence	*Mtb* residues interacting	Binding pocket(s) and involved residues
Cell wall and cell processes	**Rv0412c**	–	1–36	MTVELAHPSTEPLGSRSPAEPAHPRRWFISTTPGRI	_3_VELAHPSTE_11_	A (TYR159), B (SER66), C (TRP97), F (ASN77, TYR84)
	**Rv1183**	MmpL10	759–797	MDQVNTIIDTAKGAQPNTSLADASISMSGYPVMLRDIRD	_773_QPNTS_777_	A (LEU5), B (TYR7), C (THR70), D (HIS99, GLN156), F (ASN77)
	**Rv1811**	MgtC	207–245	MTAQALVAGVSLVGGLAVSNQAIVLLSAMIAGAGTDYAV	_2_NDY_243_	A (TYR159), B (TYR7, GLU63, SER66), *ARG62*
	**Rv1980c**	Mpt64	1–56	MRIKIFMLVTAVVLLCCSGVATAAPKTYCEELKGTDTGQA CQIQMSDPAYNINISL	_46_SDPAY_50_	E (SER147), F (ASN77, SER143, LYS146)
	**Rv2875**	Mpt70	1–34	MKVKNTIAATSFAAAAGLAALAVAVSPPAAAGDLVGP	_14_AAGLAALAV_22_	A (TYR159), B (SER66), E (GLU152), *ARG62*
	**Rv2972c**	–	1–56	MNRRLLWLSALAALALVVAYQTLGSSAGRHADEFAARAGVP TVQPGADVLAGIAV	_41_VPTVQP_46_	*ALA150*, *SER151*, D (GLN156), E (GLU152), F (LYS146)
Lipid metabolism	**Rv0214**	FadD4	272–356	SSLRRVIHAAACPQVQIKEQMIHWWGPIIDEYASSEASGSTLIT AEDWLTHPGSVGKPIQGGVHIVGADGSELPPNQPGEIYFE	_276_RVIHA_280_	F (LYS146, THR80), *GLY83*
Virulence, detoxification, adaptation	**Rv0590**	Mce2B	198–233	VNNLNTVLATTVKHQTEFDRTVDKLEVLITGLKNRA	_225_VLITGL_230_	A (SER66), B (SER24)
	**Rv1566c**	–	172–230	TPVQQAPVQPAPVQQAPVQQAPVQQAPVQQAPVQQ APVQQAPVQQAPVQQAPVQQPPPPFGTARSR	_228_RSR_230_	A (THR163), B (SER66), D (TYR159, GLN156, THR70), E (GLN156, GLN114)
Conserved hypotheticals	**Rv2578c**	–	197–238	RLALITAIRAAGLDCHVMVAPVLPQLTDSGEHLDQLLGQIAA	_221_QLTDSGE_227_	A (THR163), B (SER24, GLU63, SER66), *ARG62*
	**Rv2896c**	–	353–389	LAILEVAGLAECRDGRWRILRAGAGQAAAKGAAARLV	_370_RIL_372_	F (ASN77, TYR84)
	**Rv2990c**	–	137–168	LGKGQMLEVGDVVQVSPVRTNGMTPYLVRVLG	_137_LGKGQMLEVGDVVQVSPV_154_	A (SER66), D (GLN156), E (SER147), F (THR80, LYS146)
PE/PPE	**Rv0834c**	PE_PGRS14	32–79	AAVPTSGLLAAAGDEVSAAALFSSHGGQYQAMSAQAAAFHARFVQA	_63_QAMSAQAAAFHARF_76_	A (THR163), D (HIS155), E (GLU114), F (ASN77, THR80)
	**Rv3021c**	PPE47	41–82	LTHAVLVATNFFGINTIPIALNEADYVRMWVQAATVMSAYEA	_49_TNFFGIN_55_	D (HIS155), E (GLU152)

Bold values indicate the Mycobacterium tuberculosis locus tags (Rv numbers) corresponding to the respective genes and proteins.

#### 3.2.1 *In silico* filtering and functional classification of HLA-E-binding candidates

To evaluate candidate binding under conditions matching the *in vitro* phage display screening, computational molecular docking was executed using an isolated, β2m-free HLA-E ectodomain framework. This parallel structural audit segregated the 26 phage-enriched candidates into two distinct categories based on their spatial coordinates. A total of 12 peptides docked strictly outside the canonical peptide-binding groove (PBG). Because these candidates failed to align with native physiological coordinates, they were classified as predicted non-physiological artifacts and excluded from downstream analysis. Conversely, 14 prioritized candidates were predicted to successfully dock precisely within the canonical PBG pockets (**Supplementary Figures 2**-**S15**). Following this filtering pipeline, 14 peptides were predicted to make contacts across the six canonical HLA-E pockets. According to the Mycobrowser database, these peptides represent key functional categories: cell wall and cell processes (Rv1980c/Mpt64, Rv2875/Mpt70, Rv2972c, Rv0412c, Rv1183/MmpL10, and Rv1811/MgtC), lipid metabolism (Rv0214/FadD4), virulence/detoxification/adaptation (Rv0590/Mce2B, Rv1566c), conserved hypotheticals (Rv2578c, Rv2896c, Rv2990c), and the PE/PPE family (Rv0834c/PE_PGRS14, Rv3021c/PPE47) (**Table 1**). Many of these proteins are membrane-associated or secreted, consistent with historical descriptions of their roles in host–pathogen interactions and immune evasion.

The identified proteins range from essential metabolic enzymes to structurally diverse secreted proteins. Several candidates are critical for cell envelope integrity and lipid metabolism. Rv0412c is a conserved integral membrane protein often classified within cell wall processes (Cowley et al., 2004; Nguyen et al., 2005). Similarly, Rv1183 (MmpL10) is a large transporter implicated in mycolate transport and cell envelope biogenesis (Belardinelli et al., 2016; Ahmed et al., 2022). MmpL transporters are known to shape the outer lipidome (Ishwarlall et al., 2023). Rv1811 (MgtC) is a conserved protein involved in intramacrophage survival and adaption to magnesium limitation. The *mgtC* promoter has been shown to be expressed intracellularly, facilitating magnesium acquisition inside macrophage phagosomes (Alix et al., 2008). Rv0214 (FadD4), a fatty-acid-CoA ligase, influences cell-wall lipids (Arora et al., 2006), while Rv0590 (Mce2B) is a conserved Mce family (Sreenu et al., 2006).

The prioritized cohort also includes well-characterized secreted proteins previously described in the literature. Rv1980c (Mpt64) is a highly conserved, secreted 24-kDa antigen (major culture filtrate), deleted from most BCG strains and expressed by actively growing *Mtb* (Ahmad et al., 2019; Kılıç et al., 2025). Rv2875 (Mpt70) is a highly immunogenic, secreted protein found in *Mtb* (Windish et al., 2011; Orr et al., 2014; Ma et al., 2017; Ouyang et al., 2025). Rv1566c (RipD) is a peptidoglycan-binding protein and NlpC/p60 homolog associated with cell wall processes (Gao et al., 2006; Florio et al., 2009; Böth et al., 2014; Luan et al., 2022). The repertoire includes PE/PPE family members Rv3021c (PPE47) and Rv0834c (PE_PGRS14), which represent prominent components of the Mtb cell wall and secretome (Jain et al., 2006; Fu and Tai, 2009). Finally, several conserved hypothetical proteins were identified, including Rv2972c, a predicted exported protein (Rehren et al., 2007); Rv2578c, which features radical SAM domains (Davis et al., 2002); Rv2896c, a putative DNA-processing protein (Singhal et al., 2012); and Rv2990c (Flores-Valdez et al., 2009).

#### 3.2.2 Docking models predict engagement of HLA-E peptide-binding pockets

Following docking analysis visualization via PyMOL, the prioritized *Mtb*-derived peptides exhibited extensive predicted interfacial contact within the HLA-E antigen-binding groove. These peptides interacted across the six canonical pockets (A, B, C, D, E, and F), with localized contact analysis predicting pockets E and F as the preferred interaction sites (**Table 1**, **Supplementary Figures 2**-**S15**). Pocket F is specifically implicated in contributing to structural stability within the peptide–HLA-E model by housing primary anchor residues that bridge the peptide C-terminus to the heavy chain (Bjorkman et al., 1987; Chujoh et al., 1998; Sette and Sidney, 1999).

##### 3.2.2.1 Secreted antigens and cell wall processes

Rv1980c (Mpt64): Analysis of the docking interface suggests that the Rv1980c_1–56_ fragment establishes direct contact via its Tyr50, Asp47, and Ser46 residues with Lys146, Ser147, Ser143, and Asn77 on the HLA-E heavy chain. The interaction is predominantly mediated by Ser143, Ser147, and Asn77, which constitute the core structural architecture of the F pocket, thereby contributing to predicted complex stability and the alignment of the peptide backbone along the α-helix (Sidney et al., 2008).

Rv2875 (Mpt70): Docking analysis of the Rv2875_1–34_ fragment predicts a binding orientation where the core 9-mer region projects into pocket E, adjacent to Glu152 and Ser66. Specifically, peptide residues Ala14, Ala18, Leu20, and Val22 interact with Glu152, Ser66, Arg62, and Tyr159 of the HLA-E receptor, effectively bridging pockets A, B, and E (O'Callaghan et al., 1998).

Rv1811: The Rv1811_207–245_ fragment was predicted to bind within pockets A and B. Specifically, receptor residues Tyr7 (pocket B) and Tyr159 (pocket A) interact with peptide residue Asp242, while Glu63 and Ser66 coordinate with peptide residues Tyr243 and Thr241, respectively.

Rv0412c: Peptide residues Pro8, Glu11, and Glu4 of this candidate are predicted to engage receptor residues Tyr159, Ser66, and Trp97, respectively, to anchor the central region of the peptide sequence. The C-terminus is predicted to anchor within pocket F through the specific interaction of Asn77 with Leu5 and Tyr84 with Val3, underscoring the structural importance of the Tyr159 residue and the conserved Asn77 node.

Rv1183 (MmpL10): Docking models of the Rv1183_759–797_ region suggest a highly distributed network involving HLA-E residues Gln156, Leu5, Tyr7, His99, Thr70, and Asn77, which map to peptide residues Thr776, Gln773, Pro774, and Ser777. The intensive engagement of Asn77 and Thr70 predicts an F-pocket-centric stabilization mechanism for this transmembrane derivative.

##### 3.2.2.2 Lipid metabolism and virulence/adaptation factors

Rv0214 (FadD4): Docking configurations for Rv0214_272–356_ suggest distinct interactions linking peptide residues Arg276 and Ala280 to HLA-E groove residues Thr80 and Lys146, respectively. This predicted binding topology is consistent with an arginine-dependent anchoring mechanism within the F pocket, contributing to structural accommodation within the model (Ruibal et al., 2020; Nguyen et al., 2021).

Rv0590 (Mce2B): Interface analysis reveals that while the entire 36-mer Rv0590_198–233_ peptide was simulated, its predicted primary functional interaction footprint is localized within a core segment, _225_VLITGL_230_. Within this region, the predicted complex stability is driven by specific residue contacts in the PBG, Ser24 (pocket B) interacts directly with the peptide’s Leu230 anchor, while Ser66 (pocket A) coordinates both Val225 and Gly229. This arrangement highlights how the hydrophobic side chain of Leu230 slots into pocket B, while the surrounding residues (Val225, Leu226, Ile227, and Gly229) operate as a collective conformation that contributes to structural fit within the binding groove (Singh et al., 2016; Ruibal et al., 2020; Klepp and Y Garcia, 2022).

Rv1566c (RipD): Interface analysis of the Rv1566c docking model suggests that the Arg228 and Arg230 residues of the Rv1566c_225–230_ fragment interact with multiple residues spanning pockets A/D and D/E, respectively. Concurrently, the peptide Ser229 residue interacts with Ser66 within pocket B. The length of this sequence complies with the standard spatial requirement described for the HLA-E binding groove model (Ruibal et al., 2020).

##### 3.2.2.3 PE/PPE antigenic family members

Rv0834c (PE_PGRS14) and Rv3021c (PPE47): Peptides from this multigene family were predicted to prefer binding within pocket E. Selected fragments from both Rv0834c_32–79_ and Rv3021c_41–82_ engaged His155 within pocket D.

*PE_PGRS14*: Its interaction is predicted to be broadly across residues in pockets A, D, E, and F. F-pocket residues Asn77 and Thr80 interact with peptide residue Gln63, while receptor residues Lys146, His155, Gln114, and Thr163 coordinate with Gln68, Phe72, Arg75, and Phe76 of the peptide, respectively. This profile highlights contact with Thr80, a key F-pocket specificity residue within the structural interaction model (Nguyen et al., 2021).

*PPE47*: It exhibits a targeted network of residue interactions within the central pockets of the binding groove (**Figure 2C**). The core fragment (residues 49–55) forms localized contacts with the heavy chain and is predicted to contribute to structural accommodation through dual-pocket engagement with His155 (pocket D) and Glu152 (pocket E).

**Figure 2:**
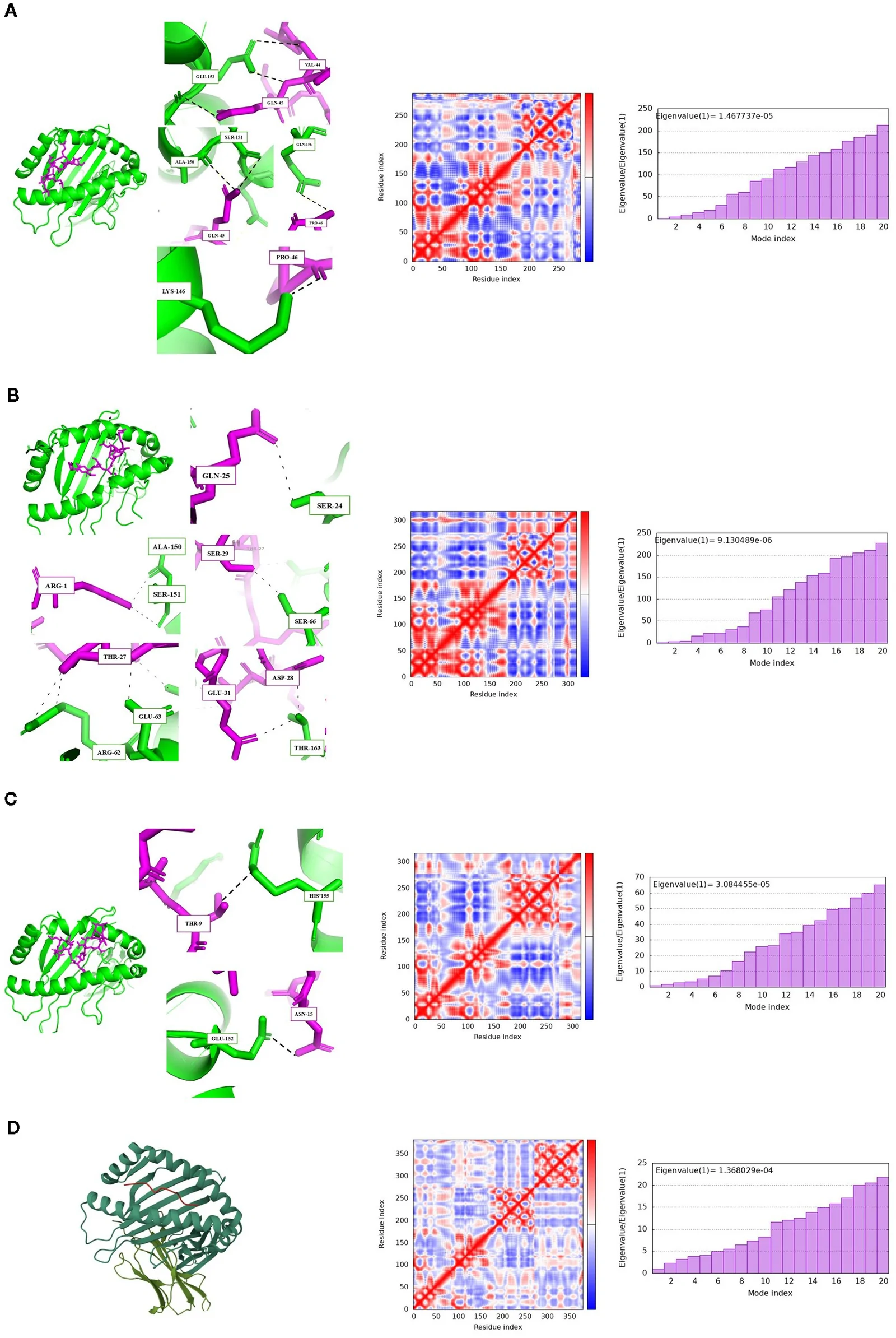
Comprehensive structural configurations and streamlined normal mode analysis (NMA) of prioritized *M. tuberculosis* (*Mtb*) peptide candidates within the HLA-E binding groove. The structural interaction properties and corresponding internal mechanics of the prioritized candidate complexes are profiled side-by-side. **(A–C)** The left column displays the three-dimensional macromolecular docking orientation (peptide in magenta, HLA-E heavy chain in green) with high-resolution structural insets mapping hydrogen bonds and electrostatic contacts (dashed lines). **(D)** The left column shows the published control Mtb44:HLA-E complex structural configuration (PDB ID: 6GH4). For all panels, the middle column presents the computed iMODS residue covariance matrix defining fully correlated (red) and anti-correlated (blue) structural motion, while the right column captures the low-frequency collective mode variance spectrum with its associated eigenvalue (*λ*). **(A)** Rv2972c complex: Displays modeled structural anchoring across receptor pockets E and F, demonstrating coordinated covariance dynamics and a low-energy eigenvalue of 1.47 × 10^-5^. **(B)** Rv2578c complex: Shows extensive pocket coordination across the HLA-E cleft, yielding predicted multiresidue interface dynamics and a low-energy collective mode profile with an eigenvalue of 9.13 × 10^-6^. **(C)** Rv3021c (PPE47) complex: Illustrates localized peptide anchoring targeted within pocket E, exhibiting aligned, correlated groove dynamics and a calculated eigenvalue of 3.08 × 10^-5^. **(D)** Mtb44 complex: Characterized control Mtb44 complex (PDB ID: 6GH4) adopted from Walters et al. (Walters et. al., 2018) used as benchmark for NMA (left) with matching coordinated internal mechanics across its covariance profile, establishing a conformationally adaptive network with a computed eigenvalue of 1.37 × 10^-4^.

##### 3.2.2.4 Conserved hypothetical proteins

Rv2972c: The Rv2972c_1–56_ fragment is predicted to interact sequentially with pockets D (Gln156), E (Glu152), and F (Lys146) via peptide residues Pro42, Val44, Gln45, and Pro46 (**Figure 2A**), respectively. This simultaneous engagement of pockets E and F points to a significant role in determining the presentation specificity of the epitope (Nguyen et al., 2021). Additionally, the peptide’s Phe41 residue coordinates with both Ala150 and Ser151 of HLA-E, which sit outside the traditionally mapped canonical pockets.

Rv2578c: For the Rv2578c_197–238_ complex, peptide residue Thr223 is predicted to form key interactions with HLA-E residues Arg62 and Glu63 located within the α-helical region of the PBG (**Figure 2B**), suggesting a potential anchoring role in structural accommodation (O'Callaghan et al., 1998; Walters et al., 2018). The predicted spatial stability of the N-terminal region is further supported by an interaction involving HLA-E residues Ser24 (pocket B), Thr163 (pocket A), and Ser66 (pocket B) to peptide residues Gln221, Asp224, and Ser225, respectively. Collectively, these interactions suggest favorable predicted peptide accommodation within the HLA-E binding groove.

Rv2896c: Docking of the Rv2896c_353–389_ fragment is predicted to focus on F-pocket engagement characterized by specific side-chain contacts: peptide residues Arg370 and Leu372 interact directly with Tyr84 and Asn77 of HLA-E, respectively. Notably, Asn77 is predicted to form a pivotal hydrogen bond with the peptide main chain, aligning with its role as an F-pocket specificity determinant (Sette and Sidney, 1999; Sidney et al., 2008; Zhu et al., 2019).

Rv2990c: The predicted interaction profile of Rv2990c_137–168_ is characterized by the simultaneous engagement of pockets A, D, E, and F. N-terminal positioning is secured via interaction between peptide residues Glu144 and Ser66, while mid-sequence stabilization is achieved by Lys139 contacting Gln156. Deep C-terminal anchoring into the groove is driven by peptide residues Val154 and Gln141 targeting Thr80 and Lys146, respectively. This predicted interaction profile is supported by Ser147, which forms dual contacts with both Leu137 and Gly138 of the peptide backbone, maximizing structural complementarity (O'Callaghan et al., 1998).

#### 3.2.3 Computational thermodynamic and mechanical modeling

##### 3.2.3.1 Scoring hierarchy of candidate epitopes

Molecular docking simulations identified a cohort of pocket-directed *Mtb* peptides with predicted global docking scores ranging from −87.49 to −44.55 kcal/mol (**Table 2**). The predicted scoring hierarchy revealed Rv2972c (−87.49 kcal/mol) as the highest-scoring candidate, followed by a cluster of favorable scoring candidates including Rv1980c (Mpt64) (−73.05 kcal/mol), Rv0214 (FadD4) (−72.71 kcal/mol), and Rv2578c (−72.27 kcal/mol).

**Table 2:** Ranking of candidate peptides based on global computational docking scores and internal coordinate mechanical stability of candidate *Mtb* complexes.

Rank	Peptide	Global docking score (kcal/mol)	Eigenvalue (*λ*)
1	**Rv2972c**	−87.49	1.46 × 10^-5^
2	**Rv1980c**	−73.05	1.09 × 10^-5^
3	**Rv0214**	−72.71	1.53 × 10^-5^
4	**Rv2578c**	−72.27	9.13 × 10^-6^
5	**Rv3021c**	−72.09	3.08 × 10^-5^
6	**Rv2875**	−69.30	1.21 × 10^-5^
7	**Rv2990c**	−61.38	1.85 × 10^-5^
8	**Rv0834c**	−57.69	1.47 × 10^-5^
9	**Rv1183**	−56.81	1.05 × 10^-5^
10	**Rv0590**	−55.48	1.33 × 10^-5^
11	**Rv0412c**	−53.03	1.68 × 10^-5^
12	**Rv2896c**	−51.20	2.79 × 10^-5^
13	**Rv1811**	−50.66	1.07 × 10^-5^
14	**Rv1566c**	−44.55	1.63 × 10^-5^
*N/A*	**Mtb44** ^a^	−	1.37 × 10^-4^

^a^ Mtb44 serves as the reference control complex; structural coordinates obtained from PDB entry 6GH4 (https://doi.org/10.2210/pdb6GH4/pdb).Bold values indicate the Mycobacterium tuberculosis locus tags (Rv numbers) corresponding to the respective genes and proteins.

Mid-tier global docking score markers were established by Rv2875 (−69.30 kcal/mol), Rv2990c (−61.38 kcal/mol), and Rv0834c (−57.69 kcal/mol), while the remaining candidates exhibited moderate baseline scores between −56.81 and −44.55 kcal/mol. These favorable computational scoring profiles across the entire selection cohort suggest complementary structural conformations within the HLA-E PBG.

##### 3.2.3.2 Structural dynamics and eigenvalue analysis

The NMA was employed to predict the mechanical stability and collective vibrational dynamics of the peptide–HLA-E complexes, using the crystallographically resolved HLA-E*01:03–Mtb44 framework (PDB ID: 6GH4) (Walters et al., 2018) as an experimental comparative control to validate the simulation baseline. The calculated eigenvalue (*γ*) served as the primary metric for structural rigidity, where higher numerical values denote increased resistance to conformational deformation (López-Blanco et al., 2014).

The benchmark control, Mtb44, exhibited the highest predicted eigenvalue in the dataset (1.37 × 10^-4^), indicating a highly rigid baseline complex (**Figure 2D**). This computational baseline is supported by the tight, overlapping alignment between the estimated NMA thermal fluctuations and the empirical PDB B-factor distributions (**Supplementary Figure 16**), suggesting that the simulation environment accurately reproduces true analysis parameters while mapping the canonical 9-mer peptide within the standard α1/α2 binding cleft (Klepp and Garcia, 2022). In contrast, the prioritized candidates identified through docking specifically Rv2578c (9.13 × 10^-6^) (**Figure 2B**), Rv1980c (1.09 × 10^-5^), and Rv2875 (1.21 × 10^-5^) demonstrated eigenvalues that were a full order of magnitude lower than the control framework (**Table 2**). This divergence indicates an elevated degree of predicted conformational flexibility, atomic deformability, and mechanical plasticity across the pocket interfaces of the newly identified *Mtb* secretory peptides, highlighting relative differences in predicted conformational flexibility compared to the Mtb44 benchmark.

## 4 Discussion

The global struggle against *M. tuberculosis* is exacerbated by the limitations of the current BCG vaccine, which fails to provide consistent, lifelong protection. This underscores an urgent need for novel immunological targets (Alsayed and Gunosewoyo, 2023; Martín et al., 2026). Most current vaccine strategies focus on classical HLA class I and II presentation; however, these pathways are hampered by extreme host genetic polymorphism, leading to variable vaccine efficacy across diverse global populations (Gillespie et al., 2025). A more critical hurdle in TB-endemic regions is the high prevalence of HIV-1 co-infection. Pathogens like HIV-1 have evolved mechanisms, such as the Nef-mediated downregulation of classical HLA-I molecules, to impair CD8^+^ T-cell surveillance. In stark contrast, the non-polymorphic HLA-E molecule remains resistant to such viral interference and is typically not downregulated in people living with HIV (Lewinsohn et al., 1998). This inherent stability, combined with its lack of polymorphism, positions HLA-E as a uniquely conserved and universal candidate for targeting T-cell-mediated immunity, particularly in high-risk and co-infected populations. Consequently, elucidating the specific *Mtb* ligands that bind this conserved receptor is essential for overcoming pathogen immune evasion and accelerating the development of next-generation vaccines (Van Stigt Thans et al., 2019; Bansal et al., 2021).

The search for novel HLA-E ligands has traditionally been limited by the receptor’s preference for canonical signal peptides, but recent advances in high-throughput proteomics and computational modeling have begun to reveal a much broader ligandome (McMurtrey et al., 2017; Bansal et al., 2021; Ruibal et al., 2022; Zhang et al., 2025). The present study contributes to this objective by employing a hybrid screening and modeling approach. By first utilizing phage display biopanning to screen an *Mtb* secretome library against recombinant HLA-E, we captured a subset of candidate peptides. This experimental screening was followed by rigorous computational docking and NMA, allowing us to prioritize candidate complexes based on global computational docking scores, geometric complementarity, and conformational flexibility.

### 4.1 Identification of *Mtb*-derived HLA-E ligands

To identify *Mtb*-derived ligands, the recombinant ectodomain of HLA-E*01:03 (UniProt: P13747) was utilized as the bait for phage display biopanning. To ensure specificity, a rigorous negative selection (preincubation) step was implemented to deplete the library of phages with non-specific affinity for the panning matrix. This experimental workflow successfully yielded an initial set of 30 *Mtb*-derived binding peptides.

To evaluate structural complementarity, we applied a computational filtering pipeline using molecular docking. While classical HLA-E presentation typically favors 8–11 amino acid (aa) nonamers, we intentionally evaluated the intact 32–85 aa secretome fragments to preserve their native conformational context and uncover nested binding motifs. Elongated peptides are well-documented to accommodate within MHC class I clefts via distinct structural adaptations, such as central conformational bulging or localized core anchoring where terminal flanking extensions protrude outward into the solvent environment while the primary anchor motif settles within the groove (Madden et al., 1993; Ruibal et al., 2022; Zhang et al., 2025). Crystallographic data confirm this unique capacity of the conserved HLA-E groove to adapt to a structurally extended pathogen ligandome (Hoare et al., 2006; Walters et al., 2018). Rather than forcing our hits into artificial nonamer boundaries, downstream refinement via FireDock (incorporating full side-chain and main-chain flexibility) mapped the precise, variable-length interaction footprints that directly anchor within the canonical pockets (**Table 1**), while flanking segments extend safely into the solvent (Zhang et al., 2025). This structural filtering narrowed our selection to 14 high-confidence sequences. These targets are predominantly derived from conserved cell wall, membrane, and secreted *Mtb* proteins essential for bacterial persistence and drug resistance (Caron et al., 2015; Jacobo-Delgado et al., 2023; Sachan et al., 2023), providing structural targets for downstream empirical investigation. Notably, our discovery of diverse HLA-E-restricted peptides from well-characterized antigens such as Mpt64, Mpt70, the membrane transporter MmpL10, and the stress-response protein Rv1811 (MgtC) highlights structural features within key functional classes of *Mtb* proteins.

In a similar vein, proteins involved in lipid transport and cellular entry such as Rv1183, the MCE-family protein Rv0590, and the peptidoglycan-binding protein RipD (Rv1566c), alongside conserved hypotheticals (Rv2578c, Rv2896c, and Rv2990c), introduce an expanded suite of novel, highly targetable candidate peptide sequences for structural evaluation.

The identification of PE/PPE family proteins highlights the structural diversity of candidate binders within this protein family. These protein families account for approximately 10% of the *Mtb* genome and represent primary coordinates for intracellular survival and evasion (Van Der Meeren et al., 2018; Zhang et al., 2019; Xie et al., 2021; Chen et al., 2025). Our isolation of pocket-directed PE/PPE candidate ligands (such as Rv0834c and PPE47) provides a structural framework for exploring candidate peptide binding characteristics.

### 4.2 Predicted site-specific pocket engagement and residue-level stabilization

Structural characterization of the HLA-E PBG reveals an architecture of distinct pockets (A–F) that is predicted to influence the binding energetics and specificity of *Mtb*-derived ligands. Our modeling suggests that the PBG exhibits significant conformational plasticity, allowing for the coordinated utilization of these pockets to accommodate diverse peptide sequences. This site-specific engagement, particularly the interplay between primary and secondary anchor pockets, appears to be a major factor behind the predicted favorable global docking scores observed during our analysis (Walters et al., 2018; Nguyen et al., 2021; Gillespie et al., 2025).

Pocket A emerged as the second most utilized site within the modeled HLA-E binding groove. The architectural configuration of this pocket is driven primarily by the Ser66 residue, which was the most frequent interactor within this site across our top-tier candidates. Furthermore, Thr163 facilitated the putative anchoring of several fragments, including those from the PE/PPE family, while Tyr159 likely acts as a critical bridge between pockets A and D, supporting structural complementarity within the model (Remesh et al., 2017; Walters et al., 2018; Nguyen et al., 2021; Hopkins et al., 2022; Walters et al., 2022).

Our computational analysis further revealed a dominant engagement of the E and F pockets within the HLA-E PBG. Pocket F is of particular importance as it traditionally houses the C-terminal primary anchor residues, suggested to support the structural configuration of the peptide–HLA complex within the computational model. In our models, pocket E appeared to coordinate peptide side chains via residues along the α2-helix, supporting overall peptide positioning and orientation (Bjorkman et al., 1987; Chujoh et al., 1998; Sette and Sidney, 1999; Remesh et al., 2017). The predicted structural features of these complexes are further reinforced by a set of key residues that appeared consistently across multiple interactions. Specifically, the F pocket interactions were consistently mediated by Asn77, Ser143, Lys146, and Thr80, while pocket E contributed to mid-peptide positioning primarily through Glu152 and Ser147 (O'Callaghan et al., 1998; Nguyen et al., 2021).

Within pocket F, Asn77 emerged as a primary determinant of ligand specificity in our models. This residue is known to form critical hydrogen bonds with the peptide backbone (Remesh et al., 2017; Zhu et al., 2019). The predicted structural characteristics of complexes with high global docking scores are further supported by interactions involving Ser143 and Ser147. Located within the α2-helix, Ser143 frequently interacts with the C-terminal residues in pocket F, contributing to deep pocket accommodation. Ser147 appears to serve a dual role, providing secondary support across the interface of pockets E and F (O'Callaghan et al., 1998; Sidney et al., 2008). The significance of these serine residues is well-documented; they are essential for maintaining the “open” conformation of the groove necessary to accommodate bulky or non-canonical *Mtb* peptides (Walters et al., 2018). Because these residues are highly conserved within HLA-E, they contribute to a consistent anchoring mechanism that supports structural accommodation of a diverse repertoire of *Mtb*-derived peptides including proteins associated with the cell wall and secretion systems (Hoare et al., 2006; Cole et al., 2014; Caccamo et al., 2015).

Complementing the F pocket, pocket E plays a vital role in stabilizing the peptide mid-section, specifically through the engagement of Glu152. As a negatively charged residue, Glu152 is predicted to secure peptides via salt bridge formation spanning the central region toward the C-terminal boundary of the E pocket. For high global docking score candidates such as Mpt70, Rv2972c, and PPE47, Glu152 provides a favorable electrostatic environment that supports localized atomic interactions within the structural model. The preferential utilization of pockets F and A, supported by a network of conserved residues such as Asn77 and Ser147, highlights key structural features underlying candidate Mtb-derived peptide binding. These findings provide a structural basis for candidate peptide prioritization, requiring downstream empirical confirmation.

### 4.3 Computational analysis of thermodynamic stability and mechanical dynamics (NMA)

The global docking score values obtained from molecular docking serve as a critical quantitative measure of the predicted structural complementarity and interaction favorability within the peptide–HLA-E complex. From a structural perspective, these computational metrics are utilized to prioritize bacterial fragments exhibiting optimal geometric fit, serving as a foundational screening step for downstream investigation (Powell et al., 2025). In the context of *Mtb* research, high global docking scores help identify which candidate sequences merit further evaluation. It is critical to note that the global docking scores derived from algorithms like PatchDock/FireDock represent relative computational scoring functions accounting for atomic desolvation, Van der Waals interactions, and electrostatic components rather than absolute experimental thermodynamic free energies (Δ*G* or *K*_d_). However, within structural modeling, these metrics serve as an effective proxy for mapping steric complementarity and optimal atomic packing within restrictive allelic grooves (Kaliamurthi et al., 2018; Waqas et al., 2020).

The observed range of global docking scores in this study is comparable to the characteristics of known canonical MHC-I binding peptides. Such low (more negative) score values indicate a high degree of structural complementarity within the PBG (Scherer et al., 2026), reflecting favorable structural complementarity within the computational model. Specifically, our prioritized candidates align with the established global docking score ranges (−70.00 to −90.00 kcal/mol) typically for canonical target peptides docked into class I HLA architectures (Kaliamurthi et al., 2018; Waqas et al., 2020). Conversely, our pipeline distinguished these pocket-directed binders from predicted non-physiological artifacts, which consistently bound to the external receptor framework and scored within the non-specific, transient range. Consequently, the computational identification of these favorable global docking score profiles narrows the scope of candidate prioritization, providing a prioritized subset of *Mtb* peptides for targeted *in vitro* and *in vivo* validation (Sethi et al., 2024). There is a clear ranking of candidate peptides based on global docking scores across the protein categories analyzed (detailed in **Table 2**). While these static geometric landscapes describe initial pocket compatibility, structural behavior can be further evaluated through dynamic modeling. To address the inherent limitations of stationary models and assess the conformational flexibility and internal dynamics of these prioritized complexes, we subsequently subjected them to internal coordinate NMA. Beyond static global docking scores, the structural behavior and flexibility of a candidate peptide can be further examined through the predicted conformational flexibility and dynamics of the peptide–MHC complex. To transition from a stationary docking profile to a dynamic assessment, we used NMA to evaluate the intrinsic flexibility and collective motions of the peptide–HLA-E complexes (Bahar et al., 2010; López-Blanco et al., 2014; Yanaka and Sugase, 2017). The eigenvalue (*λ*) provides a metric for the degree of structural deformability and conformational flexibility within the computational model.

The significance of this balance is most evident when comparing our prioritized candidates to the Mtb44 benchmark. Mtb44-HLA-E exhibited a notably higher eigenvalue (1.37 × 10^-4^), characterizing it as a relatively rigid or “stiff” complex. From a structural perspective, excessive rigidity reflects limited structural deformability within the normal mode model. In contrast, our top-tier candidates led by Rv2578c (9.13 × 10^-6^) and Mpt64 (1.09 × 10^-5^) are predicted to be an order of magnitude more flexible than the Mtb44 control. This increased conformational adaptability highlights relative differences in predicted conformational flexibility compared to the Mtb44 benchmark. The structural characteristics of these complexes were further supported by B-factor and covariance profiles. B-factor analysis indicates minimal fluctuations within the core residues of the HLA-E binding groove, suggesting that despite the predicted overall flexibility of the complex, the peptide maintains stable interactions at primary anchor points. Furthermore, covariance maps for candidates such as Rv2972c and PPE47 (Rv3021c) showed correlated motions between the peptide backbone and the HLA-E α-helices. This dynamic coupling describes coordinated atomic-level linkages within the modeled complexes.

Integrating NMA results with global docking scores provides a structural basis for prioritizing these peptide candidates. These integrated computational metrics characterize predicted structural complementarity and conformational flexibility. Consequently, these prioritized peptide candidates provide a structural foundation for downstream empirical investigation (**Table 2**). While these models offer a structural framework for candidate peptide selection, empirical validation remains necessary to evaluate biological activity.

### 4.4 Methodological boundaries and translational outlook

A primary challenge in aligning the *in vitro* phage display screening results with standard structural models stems from the architectural nature of the commercial HLA-E construct utilized during biopanning. Because this recombinant ectodomain represents a monomeric, partial HLA-E α-chain isolated from β2m, it inherently lacks the fully coordinated quaternary conformation characteristic of a native heterotrimer. To validate the structural quality of this β2m-free monomeric scaffold, we conducted an assessment using the MolProbity server. The resulting MolProbity score of 1.78 (86th percentile), alongside 0% Ramachandran outliers (refer to Section 3.2.1 and **Supplementary Table 2**), indicates that the peptide-binding pocket retains geometric and conformational features suitable for ligand interaction modeling. These data suggest that the absence of the stabilizing β2m subunit does not adversely affect the structural integrity of the binding groove utilized in our simulations. Rather than allowing this structural variance to compromise the exploratory discovery phase, this characteristic was strategically leveraged as an initial selection bottleneck and addressed across two operational fronts to systematically map and account for potential non-physiological interactions.

The *in vitro* phase employed stringent washing parameters combined with a subtractive preclearing phase during a targeted single-round biopanning workflow. This approach was intended to favor the retention of stable binders while limiting the recovery of non-specific matrix binders. Unlike massive, randomized synthetic peptide libraries (10^9^ to 10^15^ variants) that require three to five iterative rounds of selection and amplification to achieve sequence convergence, our *Mtb* F15/LAM4/KZN secretome library is a focused, low-complexity sublibrary (8 × 10^3^ variants) designed to express functional genomic fragments (Chiliza et al., 2017). Given that the HLA-E binding cleft is highly selective, relying on precise amino acid configurations to dictate peptide accommodation, the intersection of a low-complexity, pathogen-derived library with this restricted target naturally resulted in a refined elution yield. Implementing a single-round screening strategy was chosen to maintain the diversity of the target-specific immunopeptidome. As discussed by Bakhshinejad and Ghiasvand (2025) and ‘t Hoen et al. (2012), a single round of biopanning can be sufficient to isolate target-specific ligands while reducing the severe amplification biases often associated with multiround bacterial propagation. Limiting repetitive culture expansion helps mitigate library collapse and the potential loss of rare, slower-growing clones that might otherwise be outcompeted by fast-replicating variants (Derda et al., 2011; Matochko et al., 2014). This strategy enabled the recovery of a non-redundant repertoire of 36 unique clones, which were subsequently processed for downstream computational analysis.

The downstream *in silico* structural audit utilized the isolated, β2m-free HLA-E ectodomain framework to computationally mirror the physical parameters of the *in vitro* screening environment. Executing parallel molecular docking simulations on this monomeric framework provided an exploratory filter to distinguish groove-directed candidates from potential non-specific interactions. Through this workflow, 12 candidate poses that bound outside the groove were identified as potential artifacts of the heavy chain (Ravindranath et al., 2022); within the scope of this study, these data serve as a baseline for comparison. Conversely, 14 prioritized candidate sequences were modeled to anchor within the canonical PBG pockets despite the absence of the stabilizing β2m scaffold, resulting in favourable global docking score profiles (Ravindranath et al., 2023). The ability of the sequences to target the PBG under simplified conditions suggests potential geometric complementarity. This modeling indicates that these *Mtb* candidate ligands are predicted to possess structural properties capable of engaging the heavy chain in the absence of β2m, a finding that provides a structural basis for downstream empirical evaluation.

Furthermore, the use of a β2m-free HLA-E construct represents an inherent limitation of this study, as this experimental system and its corresponding monomeric model may not fully reflect the physiological HLA-E peptide-binding complex or its native structural properties. While this computational framework facilitated the prioritization of 14 candidate leads, it is subject to the inherent limitations of *in silico* approaches. Although docking and NMA provide theoretical insight into peptide–receptor engagement, these simulations rely on isolated protein components. Consequently, they do not account for the full complexity of intracellular antigen processing, such as proteasomal cleavage and TAP-mediated transport, which influence the physiological abundance of ligands on infected macrophages. Additionally, while the shorter sequence length of the Mtb44 benchmark necessitated a deviation from the standardized docking parameters used for longer candidates, its utility as a reference framework for NMA comparisons remains appropriate. Finally, while this analysis focuses on the prevalent HLA-E*01:03, the potential for peptide binding across other variants, such as HLA-E*01:01, which shares an identical PBG architecture, warrants further empirical investigation.

This study provides a computational framework for the investigation of non-classical peptide–HLA-E binding. By cataloguing these predicted binding profiles, this work serves as a foundation for future experimental studies, including *ex vivo* functional assays (such as IFN-γ ELISpot) and independent empirical validation. Collectively, these findings suggest that the HLA-E binding groove exhibits structural plasticity capable of accommodating a diverse repertoire of *Mtb*-derived peptides. These structural insights provide a baseline for prioritizing candidate peptides for downstream empirical evaluation.

## 5 Conclusion

This study established an integrated workflow combining *in vitro* phage display with *in silico* modeling to identify non-canonical *Mtb* candidate HLA-E-binding peptides. We prioritized 14 candidate sequences spanning several functional classes, including Mpt64, Mpt70, FadD4, and the PE/PPE family. Computational modeling suggests that these ligands can be accommodated within the closed-ended peptide-binding groove despite their length, utilizing precise interaction footprints in the E and F pockets while allowing flanking segments to extend toward the solvent.

Furthermore, normal mode analysis indicates that these complexes exhibit favorable global docking scores (up to −87.49 kcal/mol) and increased conformational plasticity compared to the Mtb44 benchmark. Collectively, these 14 candidates provide an exploratory structural framework to guide future empirical validation studies.

## Data Availability

All data generated or analyzed during this study are included in this published article and its supplementary information files.

## Supplementary material

Supplementary Figure 1 Ramachandran validation of the monomeric HLA-E structural model. The Ramachandran plots demonstrate the distribution of backbone dihedral angles (φ and ψ) for the generated HLA-E model. Analysis shows that 97.8% of residues fall within the favoured regions, with 100.0% in allowed regions and 0% outliers, confirming the high stereochemical quality of the peptide-binding groove.

Supplementary Figure 2 Structural modeling, site-specific pocket engagement, and mechanical dynamics of the HLA-E*01:03–Rv2972c complex. The multi-panel analysis illustrates the theoretical binding profile of the *M. tuberculosis* ligand Rv2972c. Left panels: Molecular docking overview and localized magnification of the residue-level stabilization network within the HLA-E peptide-binding groove. Specific electrostatic and hydrogen-bonding contacts are highlighted between receptor residues (green) and peptide chains (magenta), including stabilizing interactions across Gln156, Lys146, Ala150, Ser151, and Glu152. Right panels: Normal Mode Analysis (NMA) metrics tracking the dynamic plasticity of the docked complex. Plots depict the atomic deformability index, relative B-factor fluctuations, a variance covariance matrix mapping correlated/anti-correlated residue motions (red/blue spectrum), an elastic network model, and the structural variance mode index. The prioritized complex exhibits a low eigenvalue (*λ = 1.467737 x 10^-5^*), indicating a favorable, highly flexible mechanical profile optimized for potential T-cell receptor engagement.

Supplementary Figure 3 Structural modeling, site-specific pocket engagement, and mechanical dynamics of the HLA-E*01:03–Rv1980c complex. The multi-panel analysis illustrates the theoretical binding profile of the *M. tuberculosis* major immunogenic secreted antigen Rv1980c (Mpt64). Left panels: Molecular docking overview and localized magnification of the residue-level stabilization network within the HLA-E peptide-binding groove. Specific electrostatic and hydrogen-bonding contacts are highlighted between receptor residues (green) and peptide chains (magenta), including stabilizing interactions across Asn77, Lys146, Ser143, and Ser147. Right panels: Normal Mode Analysis (NMA) metrics tracking the dynamic plasticity of the docked complex. Plots depict the atomic deformability index, relative B-factor fluctuations, a variance covariance matrix mapping correlated/anti-correlated residue motions (red/blue spectrum), an elastic network model, and the structural variance mode index. The prioritized complex exhibits a low eigenvalue (*λ = 1.099865 x 10^-5^*), indicating a favorable, highly flexible mechanical profile optimized for potential T-cell receptor engagement.

Supplementary Figure 4 Structural modeling, site-specific pocket engagement, and mechanical dynamics of the HLA-E*01:03–Rv0214 complex. The multi-panel analysis illustrates the theoretical binding profile of the *M. tuberculosis* ligand Rv0214. Left panels: Molecular docking overview and localized magnification of the residue-level stabilization network within the HLA-E peptide-binding groove. Specific electrostatic and hydrogen-bonding contacts are highlighted between receptor residues (green) and peptide chains (magenta), including stabilizing interactions across Lys146, Gly83, and Thr80. Right panels: Normal Mode Analysis (NMA) metrics tracking the dynamic plasticity of the docked complex. Plots depict the atomic deformability index, relative B-factor fluctuations, a variance covariance matrix mapping correlated/anti-correlated residue motions (red/blue spectrum), an elastic network model, and the structural variance mode index. The prioritized complex exhibits a low eigenvalue (*λ = 1.538213 x 10^-5^*), indicating a favorable, highly flexible mechanical profile optimized for potential T-cell receptor engagement.

Supplementary Figure 5 Structural modeling, site-specific pocket engagement, and mechanical dynamics of the HLA-E*01:03–Rv2578c complex. The multi-panel analysis illustrates the theoretical binding profile of the *M. tuberculosis* ligand Rv2578c. Left panels: Molecular docking overview and localized magnification of the residue-level stabilization network within the HLA-E peptide-binding groove. Specific electrostatic and hydrogen-bonding contacts are highlighted between receptor residues (green) and peptide chains (magenta), including stabilizing interactions across Ser66, Thr163, Ser24, Glu63, Arg62, and Ser151. Right panels: Normal Mode Analysis (NMA) metrics tracking the dynamic plasticity of the docked complex. Plots depict the atomic deformability index, relative B-factor fluctuations, a variance covariance matrix mapping correlated/anti-correlated residue motions (red/blue spectrum), an elastic network model, and the structural variance mode index. The prioritized complex exhibits a low eigenvalue (*λ = 9.130489 x 10^-6^*), indicating a favorable, highly flexible mechanical profile optimized for potential T-cell receptor engagement.

Supplementary Figure 6 Structural modeling, site-specific pocket engagement, and mechanical dynamics of the HLA-E*01:03–Rv3021c complex. The multi-panel analysis illustrates the theoretical binding profile of the *M. tuberculosis* PPE-family virulence ligand Rv3021c (PPE47). Left panels: Molecular docking overview and localized magnification of the residue-level stabilization network within the HLA-E peptide-binding groove. Specific electrostatic and hydrogen-bonding contacts are highlighted between receptor residues (green) and peptide chains (magenta), including stabilizing interactions across His155 and Glu152. Right panels: Normal Mode Analysis (NMA) metrics tracking the dynamic plasticity of the docked complex. Plots depict the atomic deformability index, relative B-factor fluctuations, a variance covariance matrix mapping correlated/anti-correlated residue motions (red/blue spectrum), an elastic network model, and the structural variance mode index. The prioritized complex exhibits a low eigenvalue (*λ = 3.084455 x 10^-5^*), indicating a favorable, highly flexible mechanical profile optimized for potential T-cell receptor engagement.

Supplementary Figure 7 Structural modeling, site-specific pocket engagement, and mechanical dynamics of the HLA-E*01:03–Rv2875 complex. The multi-panel analysis illustrates the theoretical binding profile of the major secreted *M. tuberculosis* antigen Rv2875 (Mpt70). Left panels: Molecular docking overview and localized magnification of the residue-level stabilization network within the HLA-E peptide-binding groove. Specific electrostatic and hydrogen-bonding contacts are highlighted between receptor residues (green) and peptide chains (magenta), including stabilizing interactions across Ser66, Tyr159, Arg62, and Glu152. Right panels: Normal Mode Analysis (NMA) metrics tracking the dynamic plasticity of the docked complex. Plots depict the atomic deformability index, relative B-factor fluctuations, a variance covariance matrix mapping correlated/anti-correlated residue motions (red/blue spectrum), an elastic network model, and the structural variance mode index. The prioritized complex exhibits a low eigenvalue (*λ = 1.217506 x 10^-5^*), indicating a favorable, highly flexible mechanical profile optimized for potential T-cell receptor engagement.

Supplementary Figure 8 Structural modeling, site-specific pocket engagement, and mechanical dynamics of the HLA-E*01:03–Rv2990c complex. The multi-panel analysis illustrates the theoretical binding profile of the *M. tuberculosis* conserved hypothetical ligand Rv2990c. Left panels: Molecular docking overview and localized magnification of the residue-level stabilization network within the HLA-E peptide-binding groove. Specific electrostatic and hydrogen-bonding contacts are highlighted between receptor residues (green) and peptide chains (magenta), including stabilizing interactions across Ser66, Gln156, Lys146, Ser147, and Thr80. Right panels: Normal Mode Analysis (NMA) metrics tracking the dynamic plasticity of the docked complex. Plots depict the atomic deformability index, relative B-factor fluctuations, a variance covariance matrix mapping correlated/anti-correlated residue motions (red/blue spectrum), an elastic network model, and the structural variance mode index. The prioritized complex exhibits a low eigenvalue (*λ = 1.85 x 10^-5^*), indicating a favorable, highly flexible mechanical profile optimized for potential T-cell receptor engagement.

Supplementary Figure 9 Structural modeling, site-specific pocket engagement, and mechanical dynamics of the HLA-E*01:03–Rv0834c complex. The multi-panel analysis illustrates the theoretical binding profile of the *M. tuberculosis* PE_PGRS subfamily-associated virulence ligand Rv0834c. Left panels: Molecular docking overview and localized magnification of the residue-level stabilization network within the HLA-E peptide-binding groove. Specific electrostatic and hydrogen-bonding contacts are highlighted between receptor residues (green) and peptide chains (magenta), including stabilizing interactions across His155, Thr163, Lys146, Thr80, Asn77, and Glu114. Right panels: Normal Mode Analysis (NMA) metrics tracking the dynamic plasticity of the docked complex. Plots depict the atomic deformability index, relative B-factor fluctuations, a variance covariance matrix mapping correlated/anti-correlated residue motions (red/blue spectrum), an elastic network model, and the structural variance mode index. The prioritized complex exhibits a low eigenvalue (*λ = 1.472531 x 10^-5^*), indicating a favorable, highly flexible mechanical profile optimized for potential T-cell receptor engagement.

Supplementary Figure 10 Structural modeling, site-specific pocket engagement, and mechanical dynamics of the HLA-E*01:03–Rv1183 complex. The multi-panel analysis illustrates the theoretical binding profile of the *M. tuberculosis* lipid-transport-associated ligand Rv1183. Left panels: Molecular docking overview and localized magnification of the residue-level stabilization network within the HLA-E peptide-binding groove. Specific electrostatic and hydrogen-bonding contacts are highlighted between receptor residues (green) and peptide chains (magenta), including stabilizing interactions across Thr80, Gln156, Asn77, His99, Tyr7, and Leu5. Right panels: Normal Mode Analysis (NMA) metrics tracking the dynamic plasticity of the docked complex. Plots depict the atomic deformability index, relative B-factor fluctuations, a variance covariance matrix mapping correlated/anti-correlated residue motions (red/blue spectrum), an elastic network model, and the structural variance mode index. The prioritized complex exhibits a low eigenvalue (*λ = 1.047672 x 10^-5^*), indicating a favorable, highly flexible mechanical profile optimized for potential T-cell receptor engagement.

Supplementary Figure 11 Structural modeling, site-specific pocket engagement, and mechanical dynamics of the HLA-E*01:03–Rv0590 complex. The multi-panel analysis illustrates the theoretical binding profile of the *M. tuberculosis* virulence/adaptation-associated ligand Rv0590. Left panels: Molecular docking overview and localized magnification of the residue-level stabilization network within the HLA-E peptide-binding groove. Specific electrostatic and hydrogen-bonding contacts are highlighted between receptor residues (green) and peptide chains (magenta), including stabilizing interactions across Ser24 and Ser66. Right panels: Normal Mode Analysis (NMA) metrics tracking the dynamic plasticity of the docked complex. Plots depict the atomic deformability index, relative B-factor fluctuations, a variance covariance matrix mapping correlated/anti-correlated residue motions (red/blue spectrum), an elastic network model, and the structural variance mode index. The prioritized complex exhibits a low eigenvalue (*λ = 1.333121 x 10^-5^*), indicating a favorable, highly flexible mechanical profile optimized for potential T-cell receptor engagement.

Supplementary Figure 12 Structural modeling, site-specific pocket engagement, and mechanical dynamics of the HLA-E*01:03–Rv0412c complex. The multi-panel analysis illustrates the theoretical binding profile of the *M. tuberculosis* ligand Rv0412c. Left panels: Molecular docking overview and localized magnification of the residue-level stabilization network within the HLA-E peptide-binding groove. Specific electrostatic and hydrogen-bonding contacts are highlighted between receptor residues (green) and peptide chains (magenta), including stabilizing interactions across Trp97, Tyr84, Asn77, Tyr159, and Ser66. Right panels: Normal Mode Analysis (NMA) metrics tracking the dynamic plasticity of the docked complex. Plots depict the atomic deformability index, relative B-factor fluctuations, a variance covariance matrix mapping correlated/anti-correlated residue motions (red/blue spectrum), an elastic network model, and the structural variance mode index. The prioritized complex exhibits a low eigenvalue (*λ = 1.684477 x 10^-5^*), indicating a favorable, highly flexible mechanical profile optimized for potential T-cell receptor engagement.

Supplementary Figure 13 Structural modeling, site-specific pocket engagement, and mechanical dynamics of the HLA-E*01:03–Rv2896c complex. The multi-panel analysis illustrates the theoretical binding profile of the *M. tuberculosis* conserved hypothetical ligand Rv2896c. Left panels: Molecular docking overview and localized magnification of the residue-level stabilization network within the HLA-E peptide-binding groove. Specific electrostatic and hydrogen-bonding contacts are highlighted between receptor residues (green) and peptide chains (magenta), including stabilizing interactions across Tyr84 and Asn77. Right panels: Normal Mode Analysis (NMA) metrics tracking the dynamic plasticity of the docked complex. Plots depict the atomic deformability index, relative B-factor fluctuations, a variance covariance matrix mapping correlated/anti-correlated residue motions (red/blue spectrum), an elastic network model, and the structural variance mode index. The prioritized complex exhibits a low eigenvalue (*λ = 2.792012 x 10^-5^*), indicating a favorable, highly flexible mechanical profile optimized for potential T-cell receptor engagement.

Supplementary Figure 14 Structural modeling, site-specific pocket engagement, and mechanical dynamics of the HLA-E*01:03–Rv1811 complex. The multi-panel analysis illustrates the theoretical binding profile of the *M. tuberculosis* magnesium transporter protein Rv1811 (MgtC). Left panels: Molecular docking overview and localized magnification of the residue-level stabilization network within the HLA-E peptide-binding groove. Specific electrostatic and hydrogen-bonding contacts are highlighted between receptor residues (green) and peptide chains (magenta), including stabilizing interactions driven by key residues across Tyr159, Tyr7, Glu63, Ser66, and Arg62. Right panels: Normal Mode Analysis (NMA) metrics tracking the dynamic plasticity of the docked complex. Plots depict the atomic deformability index, relative B-factor fluctuations, a variance-covariance matrix mapping correlated/anti-correlated residue motions (red/blue spectrum), an elastic network model, and the structural variance mode index. The prioritized complex exhibits a low eigenvalue (*λ = 1.073174 x 10^-5^*), indicating a highly cooperative and stable mechanical profile optimized for potential T-cell receptor engagement.

Supplementary Figure 15 Structural modeling, site-specific pocket engagement, and mechanical dynamics of the HLA-E*01:03–Rv1566c complex. The multi-panel analysis illustrates the theoretical binding profile of the *M. tuberculosis* ligand Rv1566c. Left panels: Molecular docking overview and localized magnification of the residue-level stabilization network within the HLA-E peptide-binding groove. Specific electrostatic and hydrogen-bonding contacts are highlighted between receptor residues (green) and peptide chains (magenta), including stabilizing interactions across Ser66, Thr70, Tyr159, Thr163, Glu114, and Gln156. Right panels: Normal Mode Analysis (NMA) metrics tracking the dynamic plasticity of the docked complex. Plots depict the atomic deformability index, relative B-factor fluctuations, a variance covariance matrix mapping correlated/anti-correlated residue motions (red/blue spectrum), an elastic network model, and the structural variance mode index. The prioritized complex exhibits a low eigenvalue (*λ = 1.638551 x 10^-5^*), indicating a favorable, highly flexible mechanical profile optimized for potential T-cell receptor engagement.

Supplementary Figure 16 Normal Mode Analysis (NMA) validation metrics for the experimental HLA-E*01:03–Mtb44 control complex. Structural dynamics and collective mechanical motions were profiled for the canonical, HLA-E-restricted *M. tuberculosis* epitope Mtb44 (RLPAKAPLL; PDB ID: 6GH4) to serve as an experimental baseline benchmark for comparative deformability and flexibility profiles. Left panel: Ribbon representation of the solved HLA-E hetero-trimer complex showing the canonical anchoring of the 9-mer RLPAKAPLL peptide (red string) within the standard α1/α2 peptide-binding groove. Right panels: Normal Mode Analysis computation charts mapping the intrinsic structural plasticity of the benchmark crystal structure. Individual matrices display the atomic deformability index across the atom index, comparative B-factor distributions (contrasting NMA calculated values against empirical PDB data), a residue-to-residue cross-correlation matrix detailing coordinated structural displacements, an elastic network model, and the eigenvalue distribution mode index. The benchmark complex yields a characteristic baseline eigenvalue (*λ = 1.368029 x 10^-4^*), demonstrating the typical rigid stability profile expected of highly protective, immunogenic classical HLA-E presentation.
