# Supplementary material for: Identification of candidate HLA-E binding peptides from Mycobacterium tuberculosis using phage secretome library screening and computational modeling
Source: Front Cell Infect Microbiol. 2026 Oct 21;16:1724374. doi: 10.3389/fcimb.2026.1724374 (PMC13730909; doi:10.3389/fcimb.2026.1724374)
Supplement: Supplementary file 1 [file DataSheet1.zip › Table S1.docx]

**Table S1. Characteristics of *Mycobacterium tuberculosis* protein fragments identified from select phage display clones.**

| **Number** | **Rv no.** | **Gene Name** | **Start – End (AA)** |
| --- | --- | --- | --- |
| 1 | **Rv0214** | *fadD4* | 272–373 |
| 2 | **Rv0405c** | *pks6* | 9–81 |
| 3 | **Rv0412c** | *—* | 1–36 |
| 4 | **Rv0507** | *mmpL2* | 826–861 |
| 5 | **Rv0590** | *mce2b* | 198–233 |
| 6 | **Rv0638** | *secE1* | 1–195 |
| 7 | **Rv0805** | *—* | 133–216 |
| 8 | **Rv0834c** | *—* | 32–79 |
| 9 | **Rv1157c** | *—* | 6–73 |
| 10 | **Rv1183** | *mmpL10* | 759–797 |
| 11 | **Rv1566c** | *—* | 172–230 |
| 12 | **Rv1660** | *pks10* | 236–323 |
| 13 | **Rv1811** | *mgtC* | 207–245 |
| 14 | **Rv1820** | *ilvG* | 300–392 |
| 15 | **Rv1980c** | *mpt64* | 1–56 |
| 16 | **Rv2410c** | *—* | 42–67 |
| 17 | **Rv2578c** | *—* | 197–238 |
| 18 | **Rv2672** | *—* | 17–126 |
| 19 | **Rv2729c** | *—* | 188–213 |
| 20 | **Rv2875** | *mpt70* | 1–36 |
| 21 | **Rv2883c** | *pyrH* | 190–261 |
| 22 | **Rv2896c** | *—* | 353–389 |
| 23 | **Rv2972c** | *—* | 1–56 |
| 24 | **Rv2990c** | *—* | 137–168 |
| 25 | **Rv3021c** | *ppe47* | 41–82 |
| 26 | **Rv3177** | *—* | 235–264 |
| 27 | **Rv3365c** | *—* | 35–87 |
| 28 | **Rv3392c** | *cmaA1* | 66–87 |
| 29 | **Rv3452** | *cut4* | 13–114 |
| 30 | **Rv3475** | *—* | 147–223 |
