# Supplementary material for: Identification of candidate HLA-E binding peptides from Mycobacterium tuberculosis using phage secretome library screening and computational modeling
Source: Front Cell Infect Microbiol. 2026 Oct 21;16:1724374. doi: 10.3389/fcimb.2026.1724374 (PMC13730909; doi:10.3389/fcimb.2026.1724374)
Supplement: Supplementary file 1 [file DataSheet1.zip › Table S2.docx]

**Table S2: In Silico Structural Validation Metrics for the HLA-E Monomeric Model**

| **Metric** | **Result** | **Goal/Threshold** |
| --- | --- | --- |
| MolProbity Score | 1.78 (86th percentile) | N/A |
| Ramachandran Favoured | 97.79% | >98% (Excellent) |
| Ramachandran Outliers | 0.00% | <0.05% |
| Favored Rotamers | 100.00% | >98% |
| Clashscore (all atoms) | 16.92 | <20 (Acceptable) |
| Rama Z-Score | 0.21 ± 0.45 | < 2.0 |
| CaBLAM Outliers | 1.1% | < 1.0% |
